# Transient absorption signatures of asymmetric carrier cooling in semiconductors

**DOI:** 10.1126/sciadv.aeh6002

**Published:** 2026-10-28

**Authors:** Ivan Maliyov, Piotr Jan Kabacinski, Hemen Hosseini, Thomas F. K. Weatherley, Artur Tuktamyshev, Stefano Sanguinetti, Nicolas Grandjean, Stefano Dal Conte, Marco Bernardi, Giulio Cerullo

**Affiliations:** ^1^ Department of Applied Physics and Materials Science, and Department of Physics, California Institute of Technology, Pasadena, CA 91125, USA.; ^2^ Dipartimento di Fisica, Politecnico di Milano, Piazza L. da Vinci 32, I-20133 Milano, Italy.; ^3^ Laboratory of Advanced Semiconductors for Photonics and Electronics, Institute of Physics, Ecole Polytechnique Fédérale de Lausanne (EPFL), CH-1015 Lausanne, Switzerland.; ^4^ L-NESS and Dipartimento di Scienza dei Materiali, Università degli Studi di Milano-Bicocca, via R. Cozzi 55, I-20126 Milano, Italy.; ^5^ Istituto di Fotonica e Nanotecnologie-CNR, Piazza L. da Vinci 32, I-20133 Milano, Italy.

## Abstract

Carrier dynamics in semiconductors is central to devices for electronics, optoelectronics, energy, and quantum technologies. Following photoexcitation, electrons and holes relax on ultrafast timescales, yet resolving their individual dynamics remains challenging because widely used pump-probe spectroscopies, such as transient absorption (TA), probe the combined carrier response. Here, we disentangle the cooling dynamics of excited electrons and holes through a joint experiment-theory approach that combines high time-resolution femtosecond TA spectroscopy with quantitative first-principles simulations. We show a pronounced asymmetry in electron and hole cooling in both wurtzite and zinc-blende semiconductors, exemplified by gallium nitride (GaN) and gallium arsenide (GaAs). Guided by theory, we unravel signatures of electron and hole cooling in our femtosecond-resolved TA spectra and show that the TA response is dominated by electron cooling, which occurs on characteristic timescales on the order of 100 fs and is governed by scattering with optical phonons. In contrast, our simulations predict that hole cooling is one order of magnitude faster and produces a comparatively weak contribution to the TA response. Our study reveals the microscopic origin of this asymmetric carrier cooling, establishing it as a general feature of direct-gap wurtzite and zinc-blende semiconductors. Our work shows a predictive experiment-theory framework for understanding the individual dynamics of excited electron and hole carriers, with broad implications for conventional and quantum materials.

## INTRODUCTION

In semiconductors, photoexcitation drives electron and hole carriers out of equilibrium, with energies well above the thermal scale $k_{B}T$. After rapid carrier-carrier scattering establishes a hot Fermi-Dirac distribution, excited (or “hot”) carriers dissipate their excess energy through inelastic scattering, typically via electron-phonon (e-ph) interactions, on a subpicosecond timescale (*1*). Understanding and controlling hot carrier cooling are central to the performance of electronic and optoelectronic devices (*2*), including transistors (*3*–*6*), solar cells (*7*–*9*), nanostructured devices (*10*–*12*), light-emitting devices (*13*, *14*), sensors (*15*), and power devices (*16*).

During the cooling process, hot carriers release their excess energy and relax to the respective band edges by emitting optical and acoustic phonons. Although the qualitative mechanisms governing carrier cooling are well established (*10*), quantitatively resolving electron and hole cooling dynamics remains more challenging. Experimentally, pump-probe spectroscopies (*17*) provide direct access to ultrafast relaxation processes, but widely used techniques such as transient absorption (TA) inherently measure a combined response from both electrons and holes. As a result, separating their individual contributions has proven difficult, particularly when the relevant dynamics occurs on femtosecond timescales, where accurate measurements require high temporal resolution combined with broad probe-pulse spectral coverage to obtain an energy-resolved picture of carrier relaxation (*18*–*20*).

Recent advances in first-principles methods have enabled predictive simulations of nonequilibrium carrier dynamics, for example, using the real-time Boltzmann transport equation (rt-BTE) with e-ph interactions computed from first principles (*21*–*24*). Using this approach, Jhalani *et al.* (*21*) predicted a pronounced asymmetry between electron and hole cooling rates in wurtzite gallium nitride (GaN), with holes relaxing substantially faster than electrons due to differences in band structure and e-ph coupling. This cooling asymmetry has been proposed as a contributing factor to efficiency droop in GaN-based light-emitting diodes (*21*). However, direct experimental confirmation of asymmetric carrier cooling in GaN has remained elusive, both because of the ultrafast nature of the effect and due to the difficulty of disentangling electron and hole signals in time-domain measurements. TA measurements are ideally suited for studying carrier cooling in semiconductors, but observing the carrier cooling asymmetry in GaN requires the combination of high temporal resolution and broad spectral coverage, stretching the current capabilities of TA measurements. In addition, the interpretation of electron and hole signals in TA spectra requires guidance from theory.

In this work, we take a step toward addressing these challenges by combining sub–20-fs temporal resolution TA spectroscopy and state-of-the-art first-principles simulations of carrier cooling and the transient optical response of GaN. Our calculations play a central role in identifying the distinct features in the measured TA spectra and in separating electron and hole contributions. We show that the TA signal is dominated by electron cooling, which occurs on a 100- to 200-fs timescale and originates from electrons relaxing toward the conduction band edge. In addition, our simulations reveal weak hole-related signatures in the TA spectrum on a ∼10-fs timescale. We demonstrate that this behavior is not specific to a single material by observing analogous dynamics in gallium arsenide (GaAs). Last, we reveal the microscopic origins of the carrier cooling asymmetry, namely, the heavier effective masses and weaker phonon coupling of holes compared to electrons. Because these are generic features of direct-gap wurtzite and zinc-blende semiconductors, asymmetric carrier cooling is expected to be a general mechanism in these classes of materials.

## RESULTS

### TA measurements in GaN

We investigate the ultrafast relaxation dynamics of photoexcited carriers in thin-film GaN using broadband TA spectroscopy with high temporal resolution. The nonequilibrium optical response is measured at room temperature in transmission geometry over an energy window spanning the optical bandgap. We use sub–20-fs pump pulses with photon energies in the ultraviolet range, well above the optical gap, to generate hot carriers near the Γ valley— electrons above the conduction-band edge and holes below the valence-band edge—while remaining slightly below the threshold for direct interband transitions at the X and L valleys (*25*, *26*). Additional details of the ultrafast TA setup are provided in Materials and Methods and in note S1.

Following photoexcitation, carriers are expected to rapidly relax toward the band edges by dissipating excess energy primarily through emission of optical phonons (*21*). Our sub–20-fs pump pulses allow us to access these early-stage relaxation processes experimentally. The wide spectrum of the probe pulse and broadband detection enable high time resolution monitoring of carrier relaxation by measuring the rise time of the photobleaching signal associated with Pauli blocking of optical transitions (*27*, *28*). We note that previous optical pump-probe studies of GaN have focused on different excitation and detection regimes—typically using narrowband, two-color schemes—to investigate intervalley Γ-L scattering and determine the corresponding intervalley transition energy (*29*, *30*). To date, TA or transient reflectivity (TR) measurements of GaN with sub–100-fs temporal resolution have not been reported.

Figure 1 (A and B) shows the TA maps in GaN as a function of probe photon energy and pump-probe delay for two excitation photon energies above the optical gap, centered at 4.23 and 4.86 eV, respectively. For an incident fluence of 0.35 μJ/cm^2^ and an absorption coefficient of ∼1.4 × 10^5^ cm^−1^ at a pump photon energy of 4.86 eV (*31*), we estimate a photoexcited carrier density of 7 × 10^16^ cm^−3^. (The influence of carrier density on the TA maps is examined later in the manuscript.) Both TA maps exhibit a derivative-like spectral signature in a narrow energy range around 3.45 eV, corresponding to the absorption edge of GaN. The strong negative signal (dark blue regions in Fig. 1, A and B) is attributed to photobleaching arising from Pauli blocking of near-band-edge electronic transitions. At slightly lower probe energies, we observe a positive TA signal (dark red regions), tentatively assigned to a photoinduced red shift of the absorption edge resulting from pump-induced changes in Coulomb screening, or bandgap renormalization (*32*). This positive feature decays on a substantially faster timescale (a few hundred femtoseconds) than the bleaching signal, which dominates the nonequilibrium optical response on the picosecond timescale. At higher probe photon energies, the TA spectrum shows a significantly weaker bleaching signal that decays rapidly, within ∼200 fs.

**Fig. 1.**
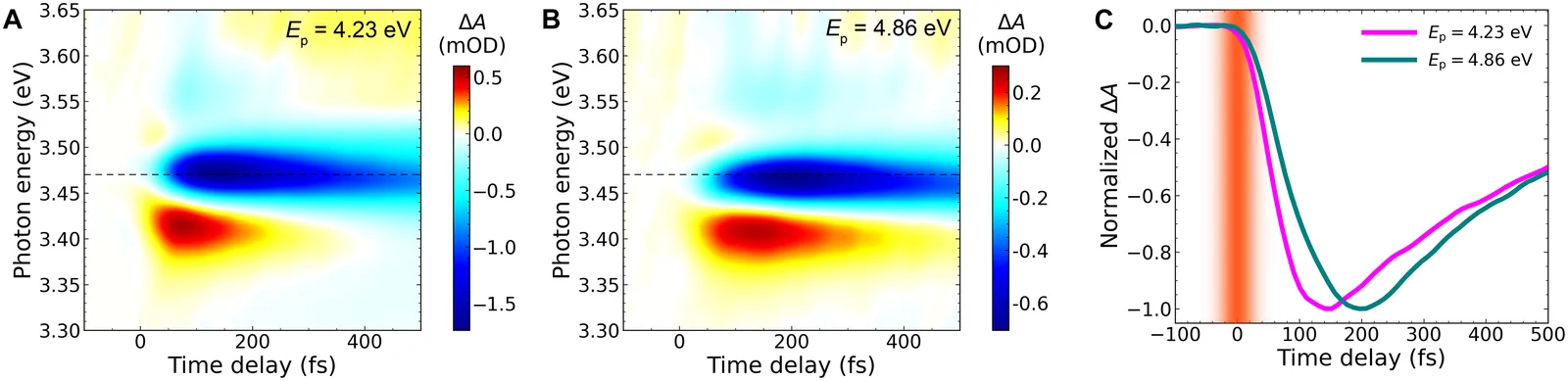
Experimental ultrafast TA maps in GaN. (**A** and **B**) TA maps, ΔA(E,t), shown as color-coded intensity as a function of pump-probe delay and probe photon energy. Results are given for two pump photon energies of 4.23 eV (A) and 4.86 eV (B). The dashed lines mark the probe energy of 3.47 eV analyzed in (C). (**C**) Normalized TA dynamics at 3.47 eV for both pump photon energies. The shaded orange region indicates the duration of the pump pulse [full width at half maximum of 20 fs (*45*)].

We analyze the dynamics of the transient response in Fig. 1C, focusing on the time evolution of the TA signal at the minimum of the photobleaching band (3.47 eV) for two pump photon energies. We find that excitation at higher photon energy leads to a slightly longer rise time of the bleaching signal. The high temporal resolution of our measurements further allows us to resolve a delay of about 20 fs in the formation of the bleaching signal at the band edge relative to that at higher probe energies.

### Modeling ultrafast carrier dynamics and TA

To interpret the experimental observations and elucidate the microscopic origin of the features observed in the TA spectra, we turn to theoretical modeling of the nonequilibrium carrier dynamics. We simulate the time-dependent evolution of photoexcited carriers using the rt-BTE, in which electron-phonon and hole-phonon scattering processes are computed fully from first principles (see Materials and Methods). This approach offers a trade-off between accuracy and computational efficiency, providing simulations that capture ultrafast carrier dynamics with femtosecond resolution over several picoseconds (*21*–*24*, *33*–*36*).

To describe hot-carrier cooling following photoexcitation, we extend the rt-BTE with e-ph scattering by introducing an explicit source term describing the optical pump pulse with finite duration

$$\frac{\partial f_{nk}\left(t\right)}{\partial t}=I^{e-\text{ph}}\left[f_{nk}\left(t\right)\right]+F_{nk}^{\text{pump}}\left(t\right)$$ (1)

where $f_{nk}\left(t\right)$ are the time-dependent electron or hole occupations for an electronic state with band index *n* and crystal momentum k and $I^{e-\text{ph}}\left[f_{nk}\left(t\right)\right]$ is the e-ph scattering integral computed from first principles (*33*, *37*); the source term $F_{nk}^{\text{pump}}\left(t\right)$ describes the pump-pulse–induced increase in the carrier population and accounts for the pump pulse energy and spectrum, pulse duration, and optical transition dipoles, with photoinduced carrier density matching the experimental value (see note S2 for details).

We perform rt-BTE simulations separately for electrons and holes to obtain their time-dependent occupations, $f_{ck}^{e}\left(t\right)$ and $f_{vk}^{h}\left(t\right)$, where *c* and *v* are conduction and valence band indices. Using these occupations, we compute the TA spectrum as a function of probe photon energy *E* and pump-probe delay *t* according to

$$\begin{array}{c} \Delta A\left(E,t\right)\propto -\sum_{\mathrm{vc}k}{|p_{\mathrm{vc}k}|}^{2}\left[f_{ck}^{e}\left(t\right)+f_{vk}^{h}\left(t\right)\right]\\ \times \delta \left[E-\left(\varepsilon_{ck}^{e}-\varepsilon_{vk}^{h}\right)\right] \end{array}$$ (2)

where $p_{\mathrm{vc}k}$ are momentum-resolved dipole matrix elements for optical transitions between valence and conduction bands, *E* is the probe photon energy, and $\varepsilon_{ck}^{e}$ and $\varepsilon_{vk}^{h}$ are electron and hole band energies, respectively. By construction, Eq. 2 allows the TA signal to be separated into distinct electron and hole contributions by selectively retaining the corresponding time-dependent occupation term, $f_{ck}^{e}\left(t\right)$ for electrons or $f_{vk}^{h}\left(t\right)$ for holes. (Note that hole occupations are defined as $f^{h}=1-f^{e}$).

### Carrier cooling signatures in TA maps

Figure 2A shows simulated TA maps in GaN for a pump photon energy of 4.23 eV. The spectral shape of the photobleaching signal and the associated cooling timescales are in good agreement with the experimental TA measurements, as is seen by comparing Fig. 2A and Fig. 1A. To characterize the spectral features associated with electron relaxation, we overlay a straight line corresponding to the simulated electron cooling trajectory on the TA map. This curve provides a visual representation of the energy loss of photoexcited electrons and quantifies their cooling rate, obtained from additional first-principles rt-BTE simulations in which carrier relaxation is modeled by initializing the carrier occupations as wave packets peaked at the pump photon energy (see note S3 and fig. S1 for details). Note that we do not provide a straight cooling trajectory for holes as the simplified analysis, which uses a constant cooling rate, does not reliably capture the dynamics near the valence-band edge.

**Fig. 2.**
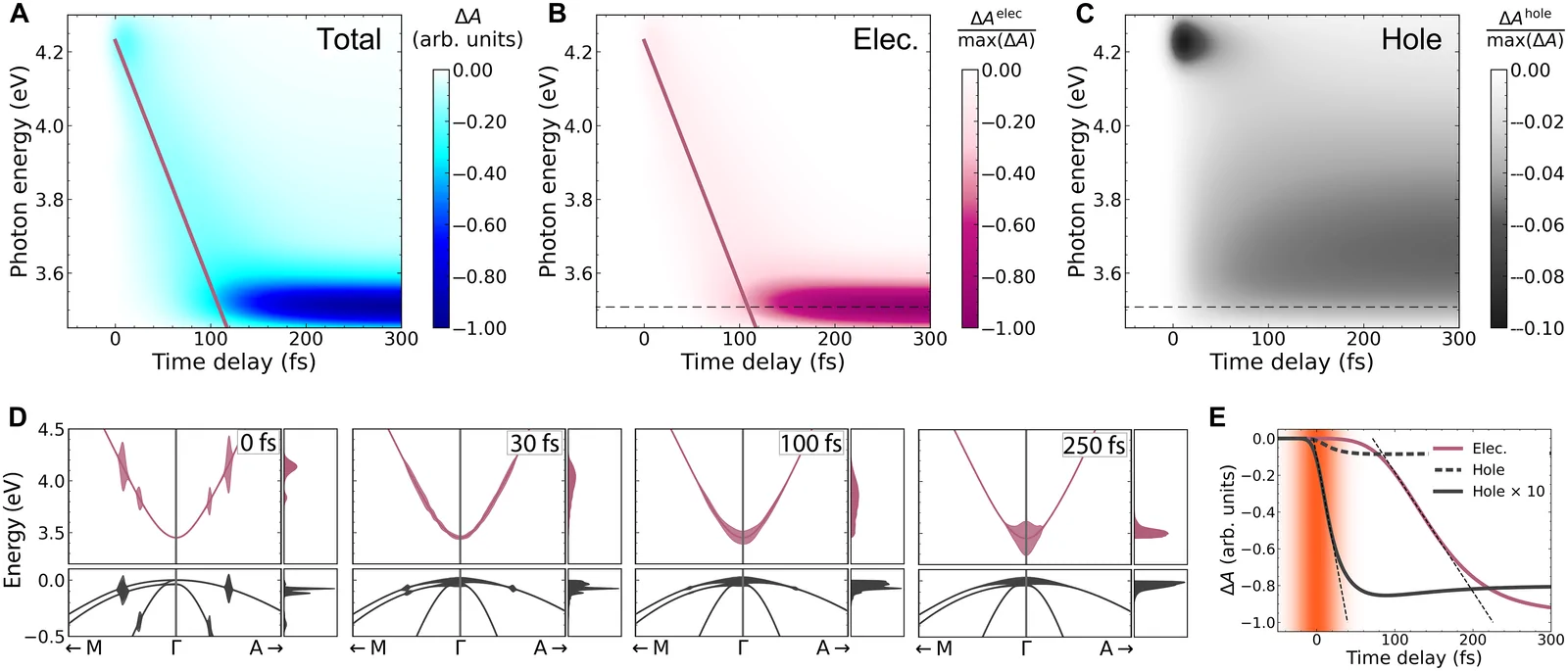
Simulated carrier dynamics and TA maps in GaN. (**A**) Normalized TA map, shown as color-coded signal (in arbitrary units) as a function of pump-probe delay time and probe photon energy at 4.23-eV pump energy. (**B** and **C**) Corresponding electron and hole contributions to the TA map, shown respectively in (B) and (C), normalized to the maximum of the signal in (A). The straight magenta line is the cooling trajectory for electrons extracted from our first-principles calculations. Note that the origin of the simulated TA maps in (A) to (C) corresponds to the bandgap, and we neglect bandgap renormalization effects in our calculations. (**D**) Time-dependent electron (magenta) and hole (gray) occupation snapshots from 0 to 250 fs, overlaid on the band structure at selected pump-probe delays. The line thickness represents the magnitude of the occupations $f_{nk}$. The side panels show Brillouin-zone integrated occupations as a function of energy for electrons (top) and holes (bottom). (**E**) Simulated electron and hole contributions to the TA signal as a function of pump-probe delay at a fixed probe energy of 3.52 eV. Both contributions are normalized to the maximum magnitude of the total TA signal. The hole contribution is shown at its original amplitude as a dashed line and, for visibility, multiplied by a factor of 10 as a solid line. The probe energy used for these temporal cuts is marked by dashed lines in (B) and (C). The black dashed lines show linear fits that help estimate the characteristic electron and hole cooling times. The shaded orange region indicates the duration of the pump pulse.

In our simulated TA maps in Fig. 2A, the signal is dominated by the electron contribution. The electron cooling trajectory closely follows the main spectral feature, starting at t=0 and E=4.2 eV and reaching a steady state at t≈250 fs and E≈3.5 eV. This behavior is further corroborated by the electron-only TA map shown in Fig. 2B, which reproduces the key features of the total transient response. In contrast, the hole contribution (Fig. 2C) exhibits a substantially broader energy distribution and reaches a steady state on a much shorter timescale, of the order of 30 fs; note that the color scale in the hole-only map is one order of magnitude smaller than that used for the total and electron-only maps. Figure 2E shows temporal cuts of the separated electron and hole contributions to the TA signal. Their distinct rise times confirm the substantially faster hole cooling, with most of the hole response developing within the first 30 fs, compared with the much slower rise (100 to 200 fs) of the electron TA signal.

The carrier distributions in momentum space shown in Fig. 2D provide additional insight into the microscopic relaxation dynamics. Following photoexcitation at t=0, electron and hole wave packets are generated with an initial energy separation equal to the pump photon energy. As relaxation proceeds through carrier-phonon scattering, both packets lose energy as they move toward their respective band edges while simultaneously broadening in momentum space. The markedly different relaxation rates of electrons and holes are evident in the occupation snapshots: Holes cool significantly faster and reach the valence-band edge on a much shorter timescale than electrons approach the conduction-band minimum. The rapid cooling of holes arises from two distinct physical mechanisms. First, GaN exhibits an intrinsic asymmetry in electron-phonon and hole-phonon coupling strengths. Because of strong hole-phonon coupling, combined with threefold valence-band degeneracy in the Γ valley, photoexcited holes lose energy more rapidly through phonon emission (*21*, *35*). From our first-principles simulations, we extract a hole cooling rate of $r_{h}=12.3$ meV/fs compared to a slower electron cooling rate of $r_{e}=5.9$ meV/fs.

Second, differences in the effective masses of electrons and holes lead to distinct initial excitation conditions. As shown in the t=0 snapshot in Fig. 2D, holes are excited closer to the valence-band edge than electrons are to the conduction-band edge. As a result, even in the hypothetical case of identical cooling rates, holes would reach the band edge earlier following photoexcitation. Both effects are captured in the analytical expression for the slope of the electron cooling trajectory, given by $-r_{e}\left(1+\frac{m_{e}^{\text{eff}}}{m_{h}^{\text{eff}}}\right)$ (see note S4 and fig. S2). For GaN, these effects together account for the order-of-magnitude difference between electron and hole cooling following optical excitation.

Another clear trend is that the TA signal is substantially larger for electrons than for holes. This behavior originates from the threefold degeneracy of the valence band at the Γ valley. The occupation of a conduction-band state with momentum k Pauli blocks optical transitions from all three valence-band states at the same k, as only a single conduction band is present near the band edge. In contrast, the occupation of a hole state blocks transitions involving only a single valence-band state. Therefore, because the densities of photoexcited electrons and holes are equal, electron relaxation produces a significantly stronger Pauli blocking signal in the TA spectrum.

### Dependence of TA on photoexcited carrier density

We next examine the dependence of the TA spectra on the photoexcited carrier concentration. Figure 3 shows the TA maps in GaN for three carrier densities, obtained from experiment (Fig. 3, A to C) and simulations (Fig. 3, D to F). For direct comparison, all maps are shown using identical energy and timescales, with a focus on the bleaching dynamics. Below the experimental TA maps in Fig. 3 (A to C), we show temporal cuts of the experimental and simulated TA signals at a fixed probe energy near the minimum of the photobleaching feature. To enable a direct comparison of the signal build-up, both the simulated and experimental temporal traces are plotted such that the TA signal reaches 5% of its maximum value at t=0 in both experiments and simulations. At carrier densities of 2.0 × 10^17^ and 4.0 × 10^17^ cm^−3^, the experimental and simulated signals reach saturation on comparable timescales of ∼100 to 200 fs and display similar early-time rise rates and overall temporal behavior. At the highest carrier density of 4.8 × 10^19^ cm^−3^, the experimental dynamics exhibits two components: an initial rise with a slope comparable to those observed at the lower densities, followed by a slower evolution that is absent from the simulated result. We tentatively attribute this slower component to electron-electron scattering, which is expected to become increasingly important at high carrier densities but is not included in our first-principles simulations.

**Fig. 3.**
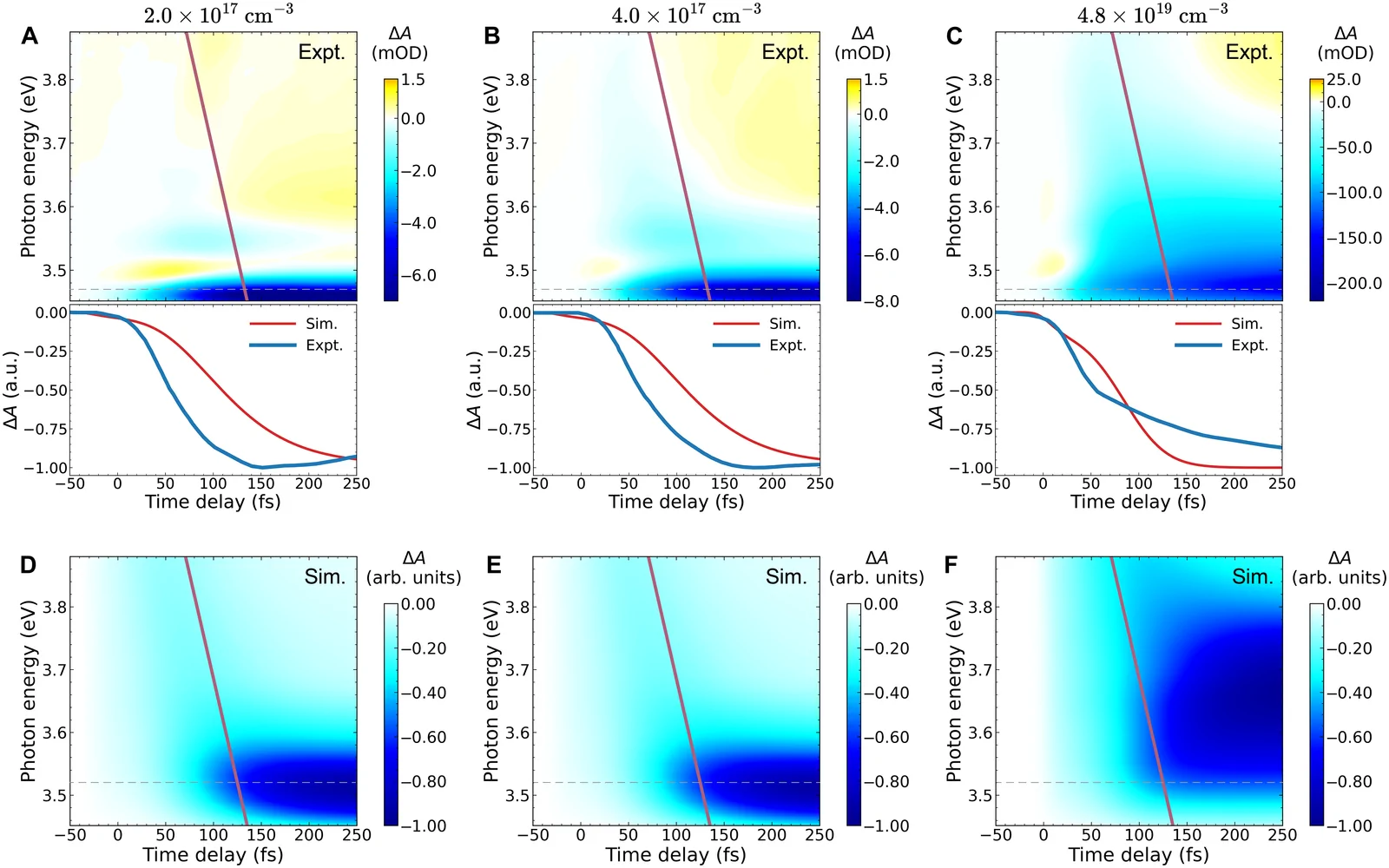
TA dependence on photoexcited carrier density in GaN. (**A** to **C**) Experimental TA maps as a function of pump-probe delay and probe photon energy for photoexcited carrier densities of (A) 2.0 × 10^17^ cm^−3^, (B) 4.0 × 10^17^ cm^−3^, and (C) 4.8 × 10^19^ cm^−3^, obtained with a pump photon energy of 4.35 eV. The panels below the experimental TA maps show the normalized TA as a function of pump-probe delay at a fixed probe energy chosen near the minimum of the saturated TA signal: 3.47 eV for the experimental curves (blue) and 3.52 eV for the simulated curves (red). (**D** to **F**) Corresponding simulated TA maps computed for the same carrier densities and pump energy. The electron cooling trajectories extracted from first-principles simulations (see note S4) are overlaid on both the experimental and simulated maps.

At the lowest carrier concentration, the cooling trace associated with electron relaxation is only weakly visible in the experimental spectrum. With increasing carrier density, this feature becomes progressively clearer and shows good agreement with the electron cooling trajectory extracted from first-principles simulations. In addition, the experimental maps exhibit an early-time low-energy tail at probe energies below ∼3.5 eV, visible at pump-probe delays *t* < 10 fs for all carrier concentrations. Although this feature is not sharply resolved in the simulated TA maps, the calculated carrier dynamics shows that hole relaxation is expected to occur on a ∼10-fs timescale. This provides indirect evidence that the low-energy tail at early times may be a signature of hole cooling.

At longer times after carrier cooling has concluded, we observe a difference between experiment and simulation in the steady-state photobleaching band (dark blue regions at *t* < 200 fs in Fig. 3), which appears narrower in the experimental maps. The width of the photobleaching band reflects the energy spread of the steady-state carrier distributions and is not captured reliably in our simulations because electron-electron (*e-e*) interactions, which become increasingly important at higher carrier densities, are not included. Nevertheless, both experiment and theory show the same trend: The width of the steady-state bleaching band increases with carrier density, reflecting the increasing energy spread of the steady-state carrier distributions. Including *e-e* scattering would modify the steady-state bleaching band in the simulated TA maps but is expected to have a negligible impact on early-time carrier cooling at the relatively low carrier densities studied here.

### Ultrafast carrier dynamics in GaAs

Having established the signatures of asymmetric electron and hole cooling in the transient optical response in GaN, we now turn to GaAs to assess the presence of asymmetric cooling and its spectroscopic signatures in a prototypical zinc-blende semiconductor. Because of the smaller bandgap in GaAs (1.42 eV compared to 3.45 eV in GaN), transmission measurements in GaAs require thin free-standing crystals or epilayers grown on transparent substrates, both of which are challenging to fabricate and handle. For this reason, we perform measurements on bulk GaAs samples in reflection geometry. Although nonequilibrium measurements generally exhibit distinct spectral responses in reflection and transmission geometries—owing to photoinduced variations of the real and imaginary parts of the dielectric function combining in nontrivial ways—their temporal evolution is expected to remain largely unchanged, as it reflects the underlying carrier dynamics rather than the specific optical detection approach.

Figure 4 compares the experimental and simulated transient optical response in GaAs. To match the color coding in the TA spectra, in Fig. 4A, we plot the negative of the experimental TR signal, −ΔR/R. With this convention, blue-negative features are associated with hot carrier cooling, while red features correspond to bandgap renormalization. In the simulations in Fig. 4B, we still analyze the TA spectra to extract the carrier cooling timescales and spectroscopic signatures.

**Fig. 4.**
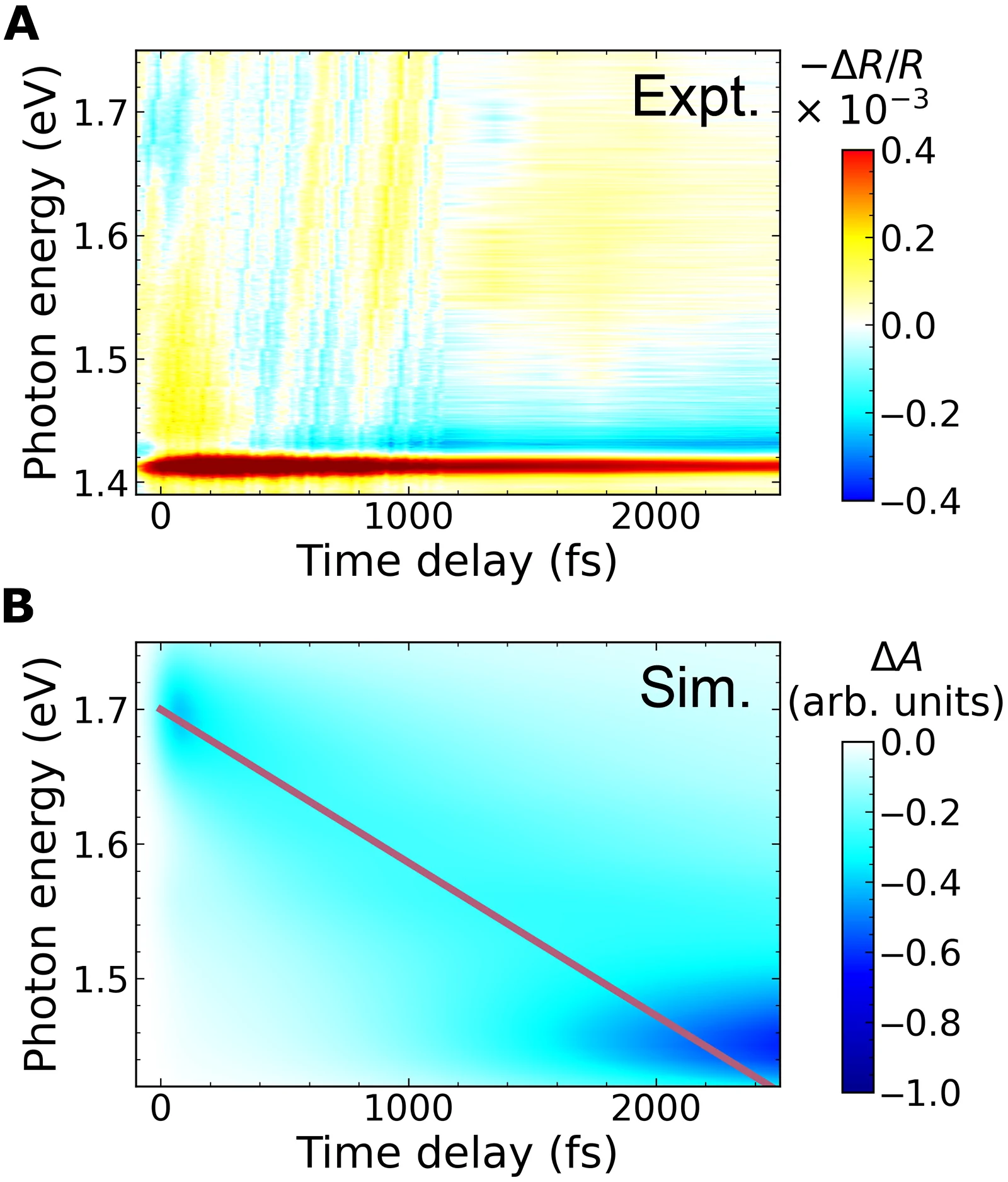
Transient optical response in GaAs. (**A**) Experimental TR map, shown as the negative of the TR signal (−ΔR/R) as a function of pump-probe delay and probe photon energy for a pump energy of 1.7 eV. (**B**) Corresponding simulated TA map for the same pump energy. The straight magenta line is the electron cooling trajectory.

In the experimental TR map, we observe a pronounced bandgap renormalization trace (red feature in Fig. 4A), while the carrier cooling signal (blue) appears at a slightly higher energy of 1.43 eV at ∼0.5 ps and reaches saturation at ∼1.0 ps. The bandgap renormalization signal occurs at zero time delay independent of the pump photon energy, whereas the carrier cooling feature exhibits progressively longer saturation times for higher pump photon energies (see note S5 and fig. S3 for TR spectra in GaAs at different pump photon energies). These observations confirm that the blue feature in the experimental TR spectrum originates from carrier cooling.

In the simulated TA spectrum shown in Fig. 4B, we observe saturation of the carrier cooling signal at ∼2.5 ps. Despite a discrepancy of about a factor of two in the cooling time, both experimental and simulated spectra exhibit clear signatures of electron relaxation and cooling. As in GaN, hole cooling in GaAs occurs on a much shorter timescale and gives rise to a weaker TA signal (see the separated carrier contributions in note S6 and fig. S4). Because of the very short timescale and weak signal, we cannot unambiguously resolve this fast hole cooling signature in the experimental TR spectra of GaAs.

Carrier cooling is substantially slower in GaAs than in GaN. Although the ratio of electron to hole effective masses in GaAs is comparable to that in GaN, the cooling rates in GaAs are significantly smaller and display a stronger asymmetry. From our simulations, we extract cooling rates of $r_{e}=0.1$ meV/fs and $r_{h}=0.7$ meV/fs. When accounting for the different carrier excitation conditions due to the different effective masses, holes excited by an optical pulse reach the band edge ∼66 times faster than electrons in GaAs. The main origin of this difference lies in the longitudinal optical (LO) phonon energies in the two materials: $\hbar \omega_{\text{LO}}∼90$ meV in GaN compared to $\hbar \omega_{\text{LO}}∼35$ meV in GaAs (*38*). Therefore, a single LO-phonon emission event removes substantially more energy from a hot carrier in GaN than in GaAs. In addition, while the hole-phonon scattering rates in GaN and GaAs are comparable, the electron scattering rates in GaAs, including contributions from LO-phonon and other scattering channels, are approximately an order of magnitude smaller than those in GaN (see note S7 and fig. S5). Together, these factors account for the substantially slower electron cooling and enhanced cooling asymmetry in GaAs.

## DISCUSSION

Our combined experimental and theoretical investigation of ultrafast carrier cooling in GaN and GaAs reveals common spectroscopic signatures in the transient optical response of both wurtzite and zinc-blende semiconductors. The TA signal is dominated by electron cooling mediated by LO-phonon emission through e-ph interactions, with relaxation to the steady state occurring on timescales ranging from ∼100 fs to several picoseconds. Guided by first-principles calculations, we identify the temporal and spectral region in which the comparatively weak hole contribution is expected to appear, on a timescale of 10 fs, although it cannot be resolved unambiguously in the experimental spectra. The pronounced asymmetry between electron and hole cooling originates from the stronger hole-phonon coupling and larger effective masses of holes, which together lead to order-of-magnitude faster hole relaxation. Theoretical modeling is essential for identifying these hole-cooling signatures, as the hole contribution to the transient optical response is comparatively weak and broadly distributed in energy. We note that this combined experiment and theory framework is transferable to other material systems in which hole signatures are more pronounced and can be distinguished clearly. Early work on GaAs quantum wells (*39*) observed a similar carrier cooling asymmetry and attributed it to hot-phonon effects, whereby excited LO phonons reheat electrons and slow their cooling. In contrast, our simulations reproduce large electron-hole cooling asymmetries in quantitative agreement with experiment without invoking coupled phonon dynamics or hot-phonon effects (*24*). This demonstrates that hot-phonon effects are not a prerequisite for observing large cooling asymmetries in bulk GaN and GaAs.

Our findings have broad implications for electronic materials and devices. On the basis of its microscopic origin, the carrier cooling asymmetry identified in this work is expected to be a general mechanism in wurtzite, zinc-blende, and possibly other classes of semiconductors. Therefore, differences in excited electron and hole dynamics could be leveraged in semiconductor-based technologies, including photovoltaics, photocatalysis, thermoelectrics, sensing, and spintronics. Beyond conventional semiconductors, other material classes—including complex oxides, organic crystals, and topological materials—are promising candidates for exhibiting similarly distinct electron and hole dynamics, owing to widely different e-ph coupling strengths, effective masses, and band dispersions of electron and hole carriers.

Our work establishes a predictive theory-experiment framework for resolving the distinct electron and hole cooling dynamics in generic materials. By advancing the microscopic understanding of nonequilibrium excited-state dynamics, this approach opens new opportunities across conventional and quantum materials. Future work will extend the modeling to capture coherent dynamics at early times and develop advanced ultrafast experimental schemes to more effectively disentangle the coupled dynamics of electrons, holes, spins, and phonons.

## MATERIALS AND METHODS

### Sample growth and preparation

#### GaN sample preparation

The 75-nm-thick GaN film is grown by metal-organic vapor phase epitaxy on a double-sided polished sapphire substrate with a 70-nm AlN nucleation layer on top. The AlN layer facilitates the high crystalline quality of the GaN layer and is optically inactive in the spectral region of the experiment because its 6-eV optical gap is much larger than that of GaN.

#### GaAs sample preparation

The 200-nm-thick GaAs layer is grown by molecular beam epitaxy on a single side polished undoped GaAs(001) substrate. The substrate temperature during the epilayer growth is 570°C, and the growth rate is 0.5 ML/s [1 ML corresponds to the site number density of the unreconstracted GaAs(001) surface: 6.26 × 10^14^ atoms/cm^−2^]. The As4 beam equivalent pressure during the growth is 8 × 10^−6^ torr measured by the ion gauge, which is equivalent to V/III ratio of ∼10. Homoepitaxial growth smooths the surface after thermally removing the native oxide layer. After the growth, the smooth surface is confirmed by the appearance of the GaAs(001)-(2x4) reconstruction monitored by the reflection high-energy electron diffraction technique.

### Computational details

Ground-state density functional theory calculations are performed using a plane-wave basis within the local density approximation (LDA) using the Quantum Espresso package (*40*). We use relaxed lattice parameters, *a* = 3.18 Å and *c* = 5.18 Å for GaN and *a* = 5.595 Å for GaAs. The plane-wave kinetic energy cutoffs are 80 Rydberg (Ry) for GaN and 72 Ry for GaAs, and scalar-relativistic norm-conserving pseudopotentials are used for both materials (*41*). The Brillouin zone is sampled with coarse k-point grids of 12 × 12 × 12 for GaN and 24 × 24 × 24 for GaAs. In the carrier dynamics and TA calculations, we use electron and hole band structures obtained from $G_{0}W_{0}$ calculations performed with the YAMBO (*42*) code on top of the LDA electronic structure, which provides improved bandgaps and carrier effective masses. Phonon dispersions and e-ph perturbation potentials are computed using density functional perturbation theory DFPT (*40*). The e-ph matrix elements are first computed on coarse grids and then interpolated to ultrafine grids using the Perturbo package (*37*) starting from Wannier functions generated with Wannier90 (*43*). The coarse grids for e-ph calculations are as follows: in GaN, 8 × 8 × 8 k-points for electrons and q-points for phonons, and in GaAs, 8 × 8 × 8 k-points for electrons and 16 × 16 × 16 q-points for phonons, using the tetrahedron integration method (*44*). We use dense grids of 180^3^ k- and 90^3^ q-points for GaN, and 160^3^ k-and 80^3^ q-points for GaAs. To compute the optical transition dipole matrix elements between valence and conduction bands, we first obtain the Kohn-Sham wave functions in Quantum Espresso on a 40 × 40 × 40 k-grid for both materials. The dipole matrix elements are then computed with YAMBO using the G-space approach (*42*) and linearly interpolated to dense grids for simulations of carrier dynamics and TA spectra. The ultrafast carrier dynamics within the rt-BTE is simulated with Perturbo using fourth-order Runge-Kutta time integration with a time step of 1 fs.

### Ultrafast optical spectroscopy

Broadband optical pump-probe measurements on GaN and GaAs were performed in transmission and reflection geometries using a home-built pump-probe setup based on a Ti:Sapphire laser. A fraction of the laser power was used to pump different home-built noncollinear optical parametric amplifiers operating in distinct spectral regions, namely, the near-IR/visible and visible/ultraviolet ranges, which were used to photoexcite the materials close to and above their bandgaps. The probe pulse consisted of a white-light supercontinuum generated by focusing either the laser fundamental or the output of a near-IR optical parametric amplifier into a CaF_2_ plate and an yttrium-aluminum-garnet plate, respectively. The estimated temporal resolution of the measurements performed on GaN and GaAs was 25 to 35 and 120 to 150 fs, respectively. Although the temporal resolution in the GaAs measurements is lower than in the GaN case, it remains fully adequate to resolve the hot-electron relaxation dynamics, which occur on timescales significantly longer than those observed in GaN. Further details about the setup are reported in the Supplementary Materials.

## Supplementary Materials

### This PDF file includes:

Notes S1 to S7 Figs. S1 to S5

## References

[1] C. V. Shank, R. L. Fork, R. F. Leheny, J. Shah, Dynamics of photoexcited GaAs band-edge absorption with subpicosecond resolution. Phys. Rev. Lett. 42, 112–115 (1979).

[2] D. K. Ferry, *Hot Carriers in Semiconductors* (IOP Publishing, 2021).

[3] E. Takeda, C. Y. Yang, C. Y.-W. Yang, A. MiuraHamada, *Hot-Carrier Effects in MOS Devices* (Academic Press, 1995).

[4] S. Vaziri, G. Lupina, C. Henkel, A. D. Smith, M. Ostling, J. Dabrowski, G. Lippert, W. Mehr, M. C. Lemme, A graphene-based hot electron transistor. Nano Lett. 13, 1435–1439 (2013). 10.1021/nl304305x 23488893

[5] T. Grasser, *Hot Carrier Degradation in Semiconductor Devices* (Springer, 2015).

[6] F. Giannazzo, G. Greco, E. Schiliro, R. Lo Nigro, I. Deretzis, A. La Magna, F. Roccaforte, F. Iucolano, S. Ravesi, E. Frayssinet, A. Michon, Y. Cordier, High-performance graphene/algan/gan schottky junctions for hot electron transistors. ACS Appl. Electron. Mater. 1, 2342–2354 (2019).

[7] R. T. Ross, A. J. Nozik, Efficiency of hot-carrier solar energy converters. J. Appl. Phys. 53, 3813–3818 (1982).

[8] D. Konig, K. Casalenuovo, Y. Takeda, G. Conibeer, J. Guillemoles, R. Patterson, L. Huang, M. Green, Hot carrier solar cells: Principles, materials and design. Phys. E Low-Dimens. Syst. Nanostructures 42, 2862–2866 (2010).

[9] M. Bernardi, D. Vigil-Fowler, J. Lischner, J. B. Neaton, S. G. Louie, Ab initio study of hot carriers in the first picosecond after sunlight absorption in silicon. Phys. Rev. Lett. 112, 257402 (2014). 10.1103/PhysRevLett.112.257402 25014830

[10] J. Shah, ed., *Hot Carriers in Semiconductor Nanostructures: Physics and Applications* (Academic Press, 1992).

[11] D. Sun, G. Aivazian, A. M. Jones, J. S. Ross, W. Yao, D. Cobden, X. Xu, Ultrafast hot-carrier-dominated photocurrent in graphene. Nat. Nanotechnol. 7, 114–118 (2012). 10.1038/nnano.2011.243 22245859

[12] Y. Dubi, Y. Sivan, “Hot” electrons in metallic nanostructures—Non-thermal carriers or heating? Light Sci. Appl. 8, 89 (2019). 10.1038/s41377-019-0199-x PMC6804576 31645933

[13] S. Saeed, E. De Jong, K. Dohnalova, T. Gregorkiewicz, Efficient optical extraction of hot-carrier energy. Nat. Commun. 5, 4665 (2014). 10.1038/ncomms5665 25116046

[14] I. Ahmed, L. Shi, H. Pasanen, P. Vivo, P. Maity, M. Hatamvand, Y. Zhan, There is plenty of room at the top: Generation of hot charge carriers and their applications in perovskite and other semiconductor-based optoelectronic devices. Light Sci. Appl. 10, 174 (2021). 10.1038/s41377-021-00609-3 PMC8408272 34465725

[15] J. Yan, M. H. Kim, J. A. Elle, A. B. Sushkov, G. S. Jenkins, H. M. Milchberg, M. S. Fuhrer, H. Drew, Dual-gated bilayer graphene hot-electron bolometer. Nat. Nanotechnol. 7, 472–478 (2012). 10.1038/nnano.2012.88 22659611

[16] M. Meneghini, C. De Santi, I. Abid, M. Buffolo, M. Cioni, R. A. Khadar, L. Nela, N. Zagni, A. Chini, F. Medjdoub, G. Meneghesso, G. Verzellesi, E. Zanoni, E. Matioli, Gan-based power devices: Physics, reliability, and perspectives. J. Appl. Phys. 130, 181101 (2021).

[17] R. Berera, R. van Grondelle, J. T. M. Kennis, Ultrafast transient absorption spectroscopy: Principles and application to photosynthetic systems. Photosynth. Res. 101, 105–118 (2009). 10.1007/s11120-009-9454-y PMC2744833 19578970

[18] C. O. Karaman, A. Y. Bykov, F. Kiani, G. Tagliabue, A. V. Zayats, Ultrafast hot-carrier dynamics in ultrathin monocrystalline gold. Nat. Commun. 15, 703 (2024). 10.1038/s41467-024-44769-3 PMC10808103 38267406

[19] Y. Luo, S. Sheng, A. Schirato, A. Martin-Jimenez, G. Della Valle, G. Cerullo, K. Kern, M. Garg, Visualizing hot carrier dynamics by nonlinear optical spectroscopy at the atomic length scale. Nat. Commun. 16, 4999 (2025). 10.1038/s41467-025-60384-2 PMC12122852 40442216

[20] G. R. Han, M. N. An, H. Jang, N. S. Han, J. Kim, K. S. Jeong, T. H. Yoon, M. Cho, In situ and real-time ultrafast spectroscopy of photoinduced reactions in perovskite nanomaterials. Nat. Commun. 16, 4956 (2025). 10.1038/s41467-025-60313-3 PMC12119852 40436875

[21] V. A. Jhalani, J.-J. Zhou, M. Bernardi, Ultrafast hot carrier dynamics in GaN and its impact on the efficiency droop. Nano Lett. 17, 5012–5019 (2017). 10.1021/acs.nanolett.7b02212 28737402

[22] X. Tong, M. Bernardi, Toward precise simulations of the coupled ultrafast dynamics of electrons and atomic vibrations in materials. Phys. Rev. Res. 3, 023072 (2021).

[23] F. Caruso, D. Novko, Ultrafast dynamics of electrons and phonons: From the two-temperature model to the time-dependent Boltzmann equation. Adv. Phys. X 7, 2095925 (2022).

[24] J. Yao, I. Maliyov, D. J. Gardner, C. S. Woodward, M. Bernardi, Advancing simulations of coupled electron and phonon nonequilibrium dynamics using adaptive and multirate time integration. npj Comput. Mater. 11, 256 (2025).

[25] J. Bourne, R. L. Jacobs, The band structure of GaN. J. Phys. C Solid State Phys. 5, 3462–3468 (1972).

[26] M. Bernardi, D. Vigil-Fowler, C. S. Ong, J. B. Neaton, S. G. Louie, Ab initio study of hot electrons in GaAs. Proc. Natl. Acad. Sci. U.S.A. 112, 5291–5296 (2015). 10.1073/pnas.1419446112 PMC4418885 25870287

[27] D. Polli, D. Brida, S. Mukamel, G. Lanzani, G. Cerullo, Effective temporal resolution in pump-probe spectroscopy with strongly chirped pulses. Phys. Rev. A 82, 053809 (2010).

[28] D. Brida, A. Tomadin, C. Manzoni, Y. J. Kim, A. Lombardo, S. Milana, R. R. Nair, K. S. Novoselov, A. C. Ferrari, G. Cerullo, M. Polini, Ultrafast collinear scattering and carrier multiplication in graphene. Nat. Commun. 4, 1987 (2013). 10.1038/ncomms2987 23770933

[29] S. Marcinkevicius, T. K. Uzdavinys, H. M. Foronda, D. A. Cohen, C. Weisbuch, J. S. Speck, Intervalley energy of GaN conduction band measured by femtosecond pump-probe spectroscopy. Phys. Rev. B 94, 235205 (2016).

[30] S. Wu, P. Geiser, J. Jun, J. Karpinski, D. Wang, R. Sobolewski, Time-resolved intervalley transitions in GaN single crystals. J. Appl. Phys. 101, 043701 (2007).

[31] J. F. Muth, J. H. Lee, I. K. Shmagin, R. M. Kolbas, H. C. Casey, B. P. Keller, U. K. Mishra, S. P. DenBaars, Absorption coefficient, energy gap, exciton binding energy, and recombination lifetime of GaN obtained from transmission measurements. Appl. Phys. Lett. 71, 2572–2574 (1997).

[32] E. A. A. Pogna, M. Marsili, D. De Fazio, S. Dal Conte, C. Manzoni, D. Sangalli, D. Yoon, A. Lombardo, A. C. Ferrari, A. Marini, G. Cerullo, D. Prezzi, Photoinduced bandgap renormalization governs the ultrafast response of single-layer MoS_2_. ACS Nano 10, 1182–1188 (2016). 10.1021/acsnano.5b06488 26691058

[33] M. Bernardi, First-principles dynamics of electrons and phonons. Eur. Phys. J. B. 89, 239 (2016).

[34] I. Maliyov, J. Park, M. Bernardi, Ab initio electron dynamics in high electric fields: Accurate prediction of velocity-field curves. Phys. Rev. B 104, L100303 (2021).

[35] I. Maliyov, J. Yin, J. Yao, C. Yang, M. Bernardi, Dynamic mode decomposition of nonequilibrium electronphonon dynamics: Accelerating the first-principles realtime Boltzmann equation. npj Comput. Mater. 10, 123 (2024).

[36] J. Sjakste, R. Sen, N. Vast, J. Saint-Martin, M. Ghanem, P. Dollfus, F. Murphy-Armando, J. Kanasaki, Ultrafast dynamics of hot carriers: Theoretical approaches based on real-time propagation of carrier distributions. J. Chem. Phys. 162, 061002 (2025). 10.1063/5.0245834 39927534

[37] J.-J. Zhou, J. Park, I.-T. Lu, I. Maliyov, X. Tong, M. Bernardi, Perturbo: A software package for ab initio electron–phonon interactions, charge transport and ultrafast dynamics. Comput. Phys. Commun. 264, 107970 (2021).

[38] P. Y. Yu, M. Cardona, *Fundamentals of Semiconductors: Physics and Materials Properties*, (Springer, ed. 4, 2010).

[39] J. Shah, A. Pinczuk, A. C. Gossard, W. Wiegmann, Energy-loss rates for hot electrons and holes in gaas quantum wells. Phys. Rev. Lett. 54, 2045–2048 (1985). 10.1103/PhysRevLett.54.2045 10031209

[40] P. Giannozzi, O. Andreussi, T. Brumme, O. Bunau, M. B. Nardelli, M. Calandra, R. Car, C. Cavazzoni, D. Ceresoli, M. Cococcioni, N. Colonna, I. Carnimeo, A. D. Corso, S. de Gironcoli, P. Delugas, R. A. D. Jr, A. Ferretti, A. Floris, G. Fratesi, G. Fugallo, R. Gebauer, U. Gerstmann, F. Giustino, T. Gorni, J. Jia, M. Kawamura, H.-Y. Ko, A. Kokalj, E. Kucukbenli, M. Lazzeri, M. Marsili, N. Marzari, F. Mauri, N. L. Nguyen, H.-V. Nguyen, A. O. de-la Roza, L. Paulatto, S. Ponce, D. Rocca, R. Sabatini, B. Santra, M. Schlipf, A. P. Seitsonen, A. Smogunov, I. Timrov, T. Thonhauser, P. Umari, N. Vast, X. Wu, S. Baroni, Advanced capabilities for materials modelling with quantum espresso. J. Phys. Condens. Matter 29, 465901 (2017). 10.1088/1361-648X/aa8f79 29064822

[41] N. Troullier, J. L. Martins, Efficient pseudopotentials for plane-wave calculations. Phys. Rev. B 43, 1993–2006 (1991). 10.1103/physrevb.43.1993 9997467

[42] D. Sangalli, A. Ferretti, H. Miranda, C. Attaccalite, I. Marri, E. Cannuccia, P. Melo, M. Marsili, F. Paleari, A. Marrazzo, G. Prandini, P. Bonfa, M. O. Atambo, F. Affinito, M. Palummo, A. Molina-Sanchez, C. Hogan, M. Gruning, D. Varsano, A. Marini, Many-body perturbation theory calculations using the yambo code. J. Phys. Condens. Matter 31, 325902 (2019). 10.1088/1361-648X/ab15d0 30943462

[43] A. A. Mostofi, J. R. Yates, G. Pizzi, Y.-S. Lee, I. Souza, D. Vanderbilt, N. Marzari, An updated version of WANNIER90: A tool for obtaining maximally-localised Wannier functions. Comput. Phys. Commun. 185, 2309–2310 (2014).

[44] P. E. Blochl, O. Jepsen, O. K. Andersen, Improved tetrahedron method for brillouin-zone integrations. Phys. Rev. B 49, 16223–16233 (1994). 10.1103/physrevb.49.16223 10010769

[45] R. Borrego-Varillas, L. Ganzer, G. Cerullo, C. Manzoni, Ultraviolet transient absorption spectrometer with sub-20-fs time resolution. Appl. Sci. 8, 989 (2018).

