# Supplementary material for: Transient absorption signatures of asymmetric carrier cooling in semiconductors
Source: Sci Adv. 2026 Oct 28;12(44):eaeh6002. doi: 10.1126/sciadv.aeh6002 (PMC13715686; doi:10.1126/sciadv.aeh6002)
Supplement: Supplementary file 1 [file sciadv.aeh6002_sm.pdf]

Science Advances
AAAS

Supplementary Materials for

Transient absorption signatures of asymmetric carrier cooling in semiconductors

Ivan Maliyov *et al.*

Corresponding author: Stefano Dal Conte, stefano.dalconte@polimi.it; Marco Bernardi, bmarco@caltech.edu

Sci. Adv. **12**, eaeh6002 (2026)
DOI: 10.1126/sciadv.aeh6002

This PDF file includes:

Notes S1 to S7
Figs. S1 to S5

SUPPLEMENTARY NOTE 1: BROADBAND OPTICAL PUMP-PROBE SPECTROSCOPY SETUP

Broadband optical pump-probe measurements on GaN and GaAs samples are carried out using a home-built apparatus, based on a Ti:Sapphire regenerative amplifier (Libra, Coherent) delivering 100-fs pulses at 1.55 eV central photon energy with a repetition rate of 1 kHz. A portion of fundamental output is used to pump a broadband visible non-collinear optical parametric amplifier (NOPA). The NOPA output pulses, spectrally spanning 1.77-2.38 eV, are compressed to sub-10-fs duration by means of chirped dielectric mirrors and subsequently frequency-doubled in a 20-μm-thick type I β-barium borate (BBO) crystal, generating broadband UV pulses with central photon energy tunable from 4.43 to 4.6 eV. The UV pulses are further compressed using an MgF_2 prism pair to nearly transform-limited 18-fs duration, and fully characterized by two-dimensional spectral interferometry. These pulses are used to photoexcite the GaN sample above the gap. The photoinduced variation in the transmittance of GaN is probed using broadband pulses spanning 1.9–3.9 eV. These probe pulses are generated via white-light continuum generation by focusing the fundamental beam into a slowly translating 2-mm-thick CaF_2 plate to avoid photodamage and ensure improved stability. The instrumental response function (IRF) of the system, depending on the probe wavelength, is estimated to be 25-35 fs.

For GaAs measurements, pump pulses are generated by a NOPA operating in the visible–near-infrared spectral range, delivering narrowband pulses ($\sim$10 nm bandwidth) tunable between 1.3 eV and 2.5 eV. The NOPA is pumped by the second harmonic of the laser (3.1 eV) generated in a BBO crystal, while the seed consists of a white-light supercontinuum produced by focusing the fundamental into a 1-mm-thick sapphire plate. A 10 nm bandpass filter is used to spectrally select the desired portion of the white-light seed prior to parametric amplification. The probe beam is a supercontinuum pulse generated by focusing the output of a near-IR optical parametric amplifier centered at 1200 nm in a 2-mm-thick sapphire plate. The IRF of the system in this configuration is estimated to be 120–150 fs.

Following the interaction with the sample, the transmitted or reflected probe are spectrally dispersed by a spectrometer (SP2150 Acton, Princeton Instruments) and detected using a linear image sensor driven by a custom-built electronic board (Stresing Entwicklungsburo GmbH). For each probe wavelength, transient absorption and reflectivity signals are recorded as a function of the pump-probe delay.

SUPPLEMENTARY NOTE 2: BOLTZMANN TRANSPORT EQUATION WITH PUMP PULSE CARRIER EXCITATION

To simulate ultrafast carrier dynamics, use the real-time Boltzmann transport equation (rt-BTE) with an optical pump term:

$$\frac{\partial f_{n\mathbf{k}}(t)}{\partial t} = \mathcal{I}^{e-\mathrm{ph}}[f_{n\mathbf{k}}(t)] + F^{\mathrm{pump}}_{n\mathbf{k}}(t). \tag{1}$$

The pump pulse term in this equation generates excited carriers. For excited electrons, this term reads:

$$F^{\mathrm{pump}}_{c\mathbf{k}}(t) = I_p(t) \sum_v |\mathbf{p}_{vc\mathbf{k}}|^2 \exp\Big\{-\frac{1}{2\sigma^2}\big(E_p - (\varepsilon^e_{c\mathbf{k}} - \varepsilon^h_{v\mathbf{k}})\big)^2\Big\}, \tag{2}$$

where v and c label conduction and valence bands, respectively, E_p is the pump-pulse photon energy and σ its energy broadening, $\mathbf{p}_{vc\mathbf{k}}$ are interband optical transition dipoles, while $\varepsilon^e_{c\mathbf{k}}$ and $\varepsilon^h_{v\mathbf{k}}$ are electron and hole band energies. For holes, the pump pulse term $F_{v\mathbf{k}}$ has the same expression, but with the summation running over electron bands c. The pump pulse intensity $I_p(t)$ is modeled as a Gaussian in time. To match the experimental conditions, we use a pulse duration with FWHM of 20 fs for GaN and 120 fs for GaAs. The maximum value of $I_p(t)$ is chosen to match the experimental photoinjected carrier density, with values of 7×10^{16} cm^{-3} for the GaN TA spectrum in Fig. 2 of main text, and values equal to the carrier densities reported in Fig. 3 of main text for additional measurements in GaN. The carrier density for GaAs is 1.9×10^{16} cm^{-3}. The energy broadening σ matches the experimental broadening of the pump pulse, with FWHM values of 40 meV for GaN and 15 meV for GaAs.

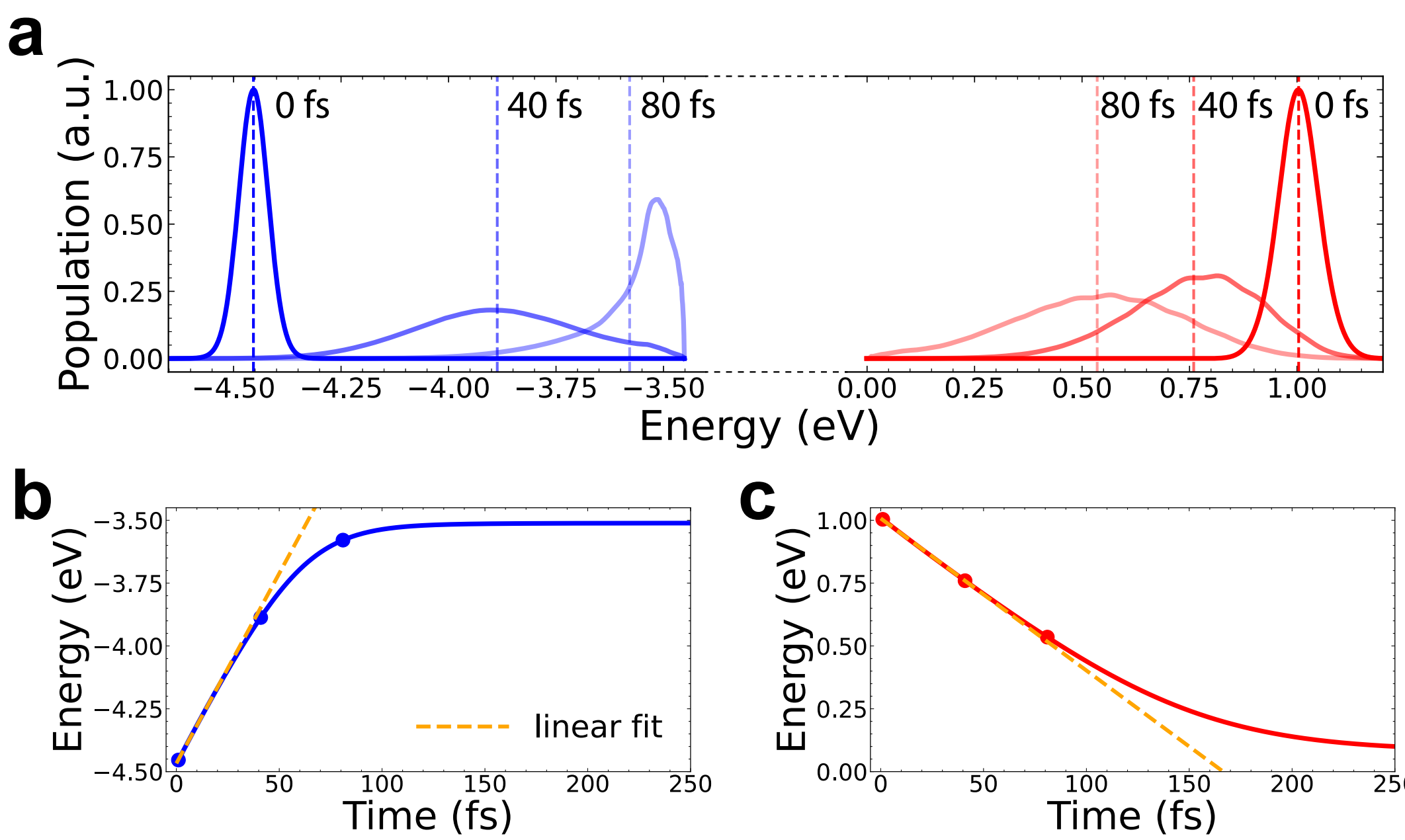

Fig. S1. **Calculation of carrier cooling rates from ultrafast dynamics simulations.** **a** Momentum-averaged carrier populations in GaN, $\bar{f}(E,t)$, for holes (blue) and electrons (red) as a function of energy at simulation times of 0, 40, and 80 fs. To obtain the cooling rate, the initial carrier distributions are taken to be Gaussian energy distributions with 1 eV excess energy relative to the respective band edges. **b**, **c** Energy of the peak of the carrier distributions for holes (**b**) and electrons (**c**) as a function of time. The dashed orange lines show linear fits to the early-time dynamics used to extract the cooling rates. Circles on top of the curves correspond to times 0, 40, and 80 fs.

SUPPLEMENTARY NOTE 3: FIRST-PRINCIPLES CALCULATIONS OF HOT CARRIER COOLING RATES

We describe the procedure to extract the electron and hole cooling rates from the first-principles ultrafast dynamics simulations. To compute the intrinsic carrier cooling rates, instead of exciting a system with an optical pump pulse, we follow the approach used in Jhalani *et al.* (*22*) and excite the electron and hole carriers with a 1-eV excess energy relative to the respective band edges. We then time-step rt-BTE (Eq. 1 without the pump-pulse term) to compute the carrier relaxation toward the band edges due to the electron-phonon (e-ph) interactions. From the time-dependent occupation function, $f_{n\mathbf{k}}(t)$, we compute the momentum-averaged, energy dependent carrier populations:

$$\bar{f}(E,t) = \sum_{n\mathbf{k}} f_{n\mathbf{k}}(t)\,\delta(\varepsilon_{n\mathbf{k}} - E). \tag{3}$$

To accurately sum over the Brillouin zone, we employ the tetrahedron integration method (*45*) as implemented in the Perturbo code (*38*).

Figure S1a shows the momentum-averaged carrier populations for holes (blue) and electrons (red) at time snapshots $t = 0$, 40 fs, and 80 fs. The average energy of the populations as a function of time, $\bar{E}(t) = \sum_E \bar{f}(E,t)E$, is shown in Fig. S1b,c for holes and electrons, respectively. At early times after excitation, we find that both electron and hole populations lose their energy linearly in time, with a trend $\bar{E}(t) = E_0 - r_{e/h} \times t$, where $r_{e/h}$ is the electron or hole cooling rate, and E_0 is the initial average energy. Table S1 gives the computed cooling rates for GaN and GaAs extracted from a linear fit of the carrier populations at early times in these simulations (Fig. S1b,c) . The carrier effective masses and the cooling asymmetry r_h/r_e are also reported in Table S1.

Material	$m_e^{\rm eff}$ (m_0)	$m_{hh}^{\rm eff}$ (m_0)	$m_{lh}^{\rm eff}$ (m_0)	r_e (meV/fs)	r_h (meV/fs)	asymmetry r_h/r_e
GaN	0.2	1.6	0.15	5.9	12.3	2.1
GaAs	0.07	0.67	0.0619	0.10	0.69	6.9

Table. S1. Effective masses in the Γ-valley and relaxation rates of electrons and holes in GaN and GaAs from *ab initio*. For holes, both heavy and light hole effective masses, $m_{hh}^{\rm eff}$ and $m_{lh}^{\rm eff}$ are specified.

SUPPLEMENTARY NOTE 4: ELECTRON AND HOLE COOLING LINES IN THE TA SPECTRA

We describe the computation of the cooling lines (or cooling "trajectories") overlaid to the TA spectra in main text. Those lines are not obtained by fitting the experimental or first-principles results, but rather they are obtained from separate calculations. To compute the cooling lines, we approximate the band dispersions as parabolic near the band edges, and assume a constant carrier cooling rate. The parabolic approximation gives the following form for the band structure of electrons and holes:

$$\varepsilon^e(k) = \alpha_e k^2 + E_c \tag{4a}$$

$$\varepsilon^h(k) = -\alpha_h k^2 + E_v, \tag{4b}$$

where E_c is the conduction band minimum and E_v is the valence band maximum, and k is the absolute value of momentum relative to the band edge. Here, the coefficients α_e and α_h are inversely proportional to the electron and hole effective masses, $m_e^{\rm eff}$ and $m_h^{\rm eff}$.

With an optical excitation of energy E_p, electrons and holes are initially excited by a vertical optical transition at the same crystal momentum k_0 (see Fig. S2). The corresponding initial transition energy is defined as

$$E_p = \varepsilon^e(k_0) - \varepsilon^h(k_0). \tag{5}$$

Within the parabolic band approximation, we then obtain:

$$E_p = E_c - E_v + (\alpha_e + \alpha_h)k_0^2. \tag{6}$$

Assuming constant cooling rates, the energies of the electron and hole packets evolve linearly in time as:

$$E^e_{\rm packet}(t) = E_0^e - r_e t \tag{7a}$$

$$E^h_{\rm packet}(t) = E_0^h + r_h t \quad , \tag{7b}$$

where r_e and r_h are electron and hole cooling rates extracted from first-principles ultrafast dynamics simulations (see Section). The initial packet energies correspond to the same initial momentum k_0:

$$E_0^e = \varepsilon^e(k_0), \qquad E_0^h = \varepsilon^h(k_0). \tag{8}$$

Using equations 4a and 4b, we derive the time dependence of the momenta of the packets:

$$k^e(t)^2 = \frac{1}{\alpha_e}\big(E_0^e - E_c - r_e t\big), \tag{9a}$$

$$k^h(t)^2 = \frac{1}{\alpha_h}\big(E_v - E_0^h - r_h t\big). \tag{9b}$$

Focusing on the electron packet, as a result of electron cooling due to e–ph interactions, the vertical transition energy evaluated at the electron packet momentum evolves as (see Fig. S2):

$$\Delta E^e(t) = \varepsilon^e\big(k^e(t)\big) - \varepsilon^h\big(k^e(t)\big). \tag{10}$$

Using equations 4 and the relation $\alpha_h/\alpha_e = m_e^{\rm eff}/m_h^{\rm eff}$, we obtain:

$$\Delta E^e(t) = E_p - r_e t\Big(1 + \frac{m_e^{\rm eff}}{m_h^{\rm eff}}\Big). \tag{11}$$

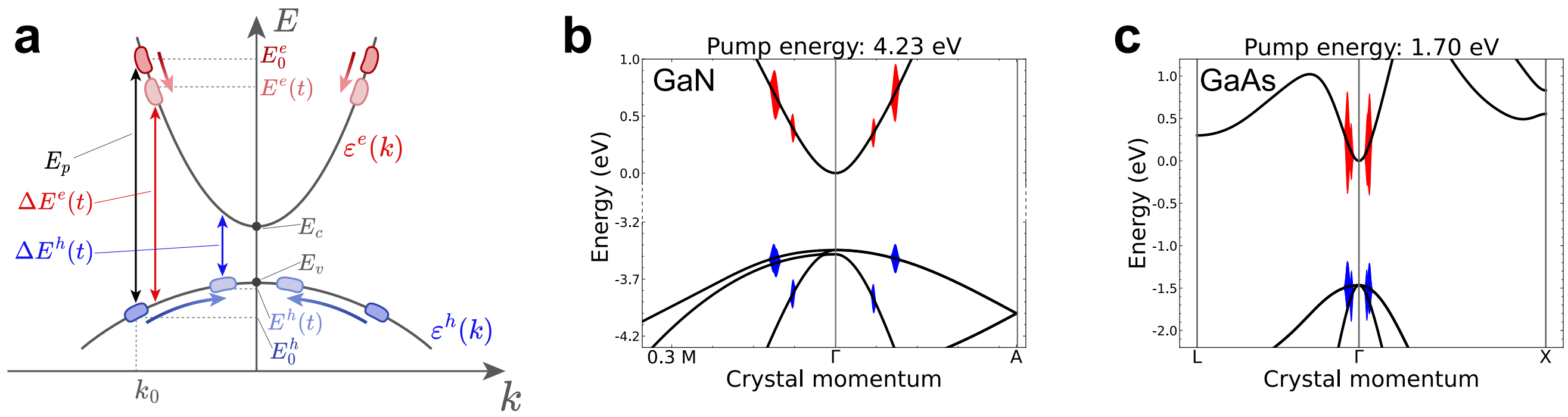

Fig. S2. **Excited electron and hole carrier packets in GaN and GaAs.** **a** Schematic of the electron and hole cooling model. **b**, **c** Band structure and initial electron (red) and hole (blue) occupations in GaN and GaAs.

This expression describes a straight line with a negative slope in the time-energy plane and corresponds to the electron cooling traces in the TA maps shown in the main text.

We derive corresponding expressions for the time-dependent transition energy for the hole packet. To evaluate the vertical transition at the momentum of the hole packet, we replace $k^e(t)$ with $k^h(t)$ and use equation 9b:

$$\Delta E^h(t) = \varepsilon^e\big(k^h(t)\big) - \varepsilon^h\big(k^h(t)\big). \tag{12}$$

Using equations 4a and 4b, together with the relation $\alpha_e/\alpha_h = m_h^{\text{eff}}/m_e^{\text{eff}}$, the resulting expression for the hole cooling line in the TA maps is

$$\Delta E^h(t) = E_p - r_h t\Big(1 + \frac{m_h^{\text{eff}}}{m_e^{\text{eff}}}\Big). \tag{13}$$

Note that the electron and hole cooling lines are meaningful only at early times after photoexcitation, consistent with their use in the main text, and they do not describe carrier dynamics and saturation at steady state.

SUPPLEMENTARY NOTE 5: PUMP PHOTON ENERGY DEPENDENCE OF TRANSIENT RESPONSE IN GaAs

Figure S3a–c shows different transient reflectivity maps measured as a function of excitation energy in GaAs. The map measured with a pump resonant with the optical bandgap is dominated by an instantaneous signal at zero delay time, characterized by a derivative-like shape and related to the pump-induced bandgap renormalization caused by the change in Coulomb screening. As the pump photon energy increases, a clear contribution from carrier cooling (blue trace centered at ∼1.43 eV) becomes visible. This contribution is characterized by a formation time on the order of picoseconds, which increases with the excess energy with which the carriers are optically injected above the bandgap. Panel S3d shows the temporal dynamics at the probe energies corresponding to the hot carrier relaxation signal. The negative peaks associated with hot carrier relaxation occur at longer times for larger pump energies.

SUPPLEMENTARY NOTE 6: ELECTRON AND HOLE CONTRIBUTION TO THE TA SPECTRUM IN GaAs

To further analyze the hot carrier cooling dynamics in GaAs, we show in Fig. S4 below the electron and hole contributions to the TA spectrum of GaAs, while the total TA map is shown in the main text.

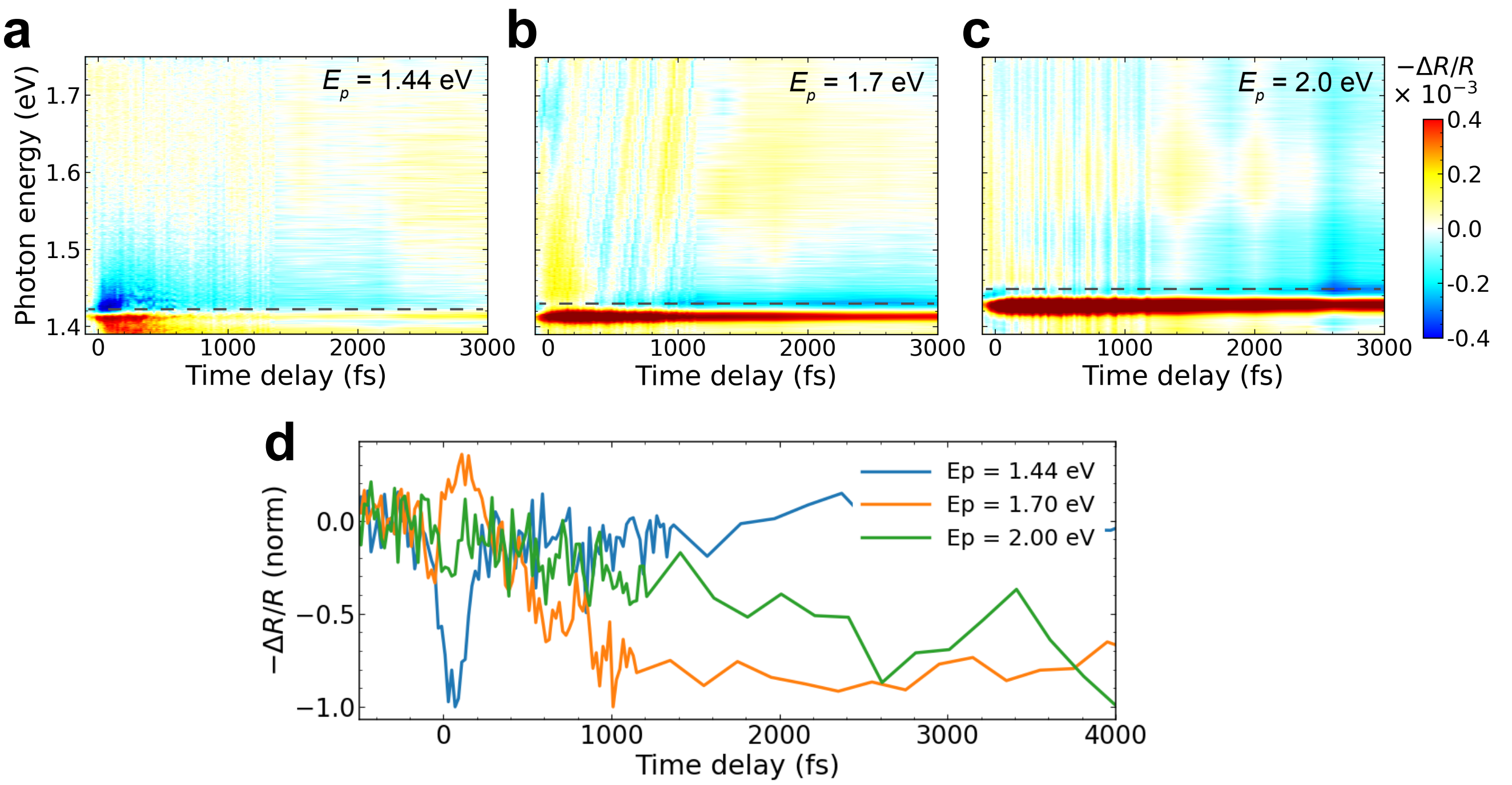

Fig. S3. **Transient reflectivity dependence on pump photon energy in GaAs. a, b, c** Experimental inverted transient reflectivity ($-\Delta R/R$) maps as a function of pump–probe delay and probe photon energy for pump energies of 1.44 eV (**a**), 1.7 eV (**b**), and 2.0 eV (**c**). **d** Temporal dynamics for the three pump energies. Normalized inverted transient reflectivity as a function of time at the probe energies corresponding to the hot carrier signal: 1.42, 1.43, and 1.45 eV for the pump energies of 1.44, 1.7, and 2.0 eV, respectively. These probe energies are indicated by horizontal dashed lines in panels **a**, **b**, and **c**.

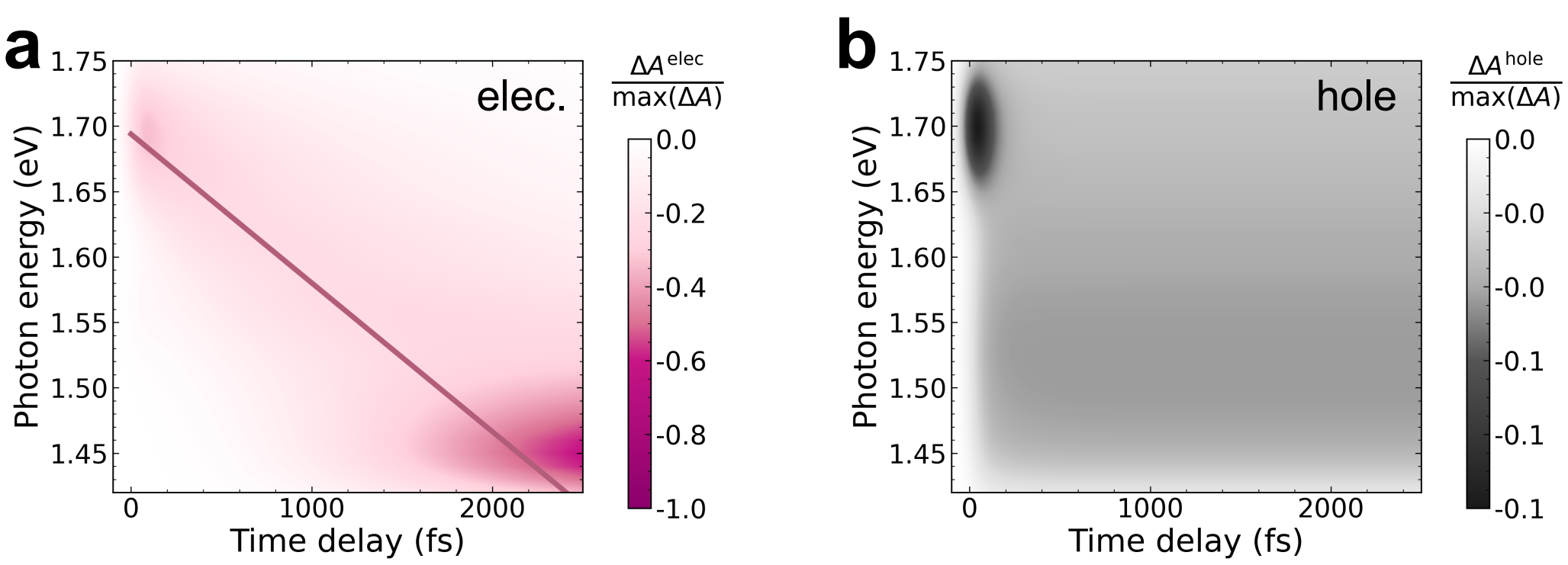

Fig. S4. **Carrier contributions to the ultrafast TA spectrum in GaAs. a, b** Simulated electron (**a**) and hole (**b**) TA maps. The electron cooling line in magenta is shown on top of the spectra.

SUPPLEMENTARY NOTE 7: CARRIER-PHONON SCATTERING RATES IN GaN AND GaAs

To further analyze the carrier–phonon scattering mechanisms underlying the hot carrier cooling dynamics in GaN and GaAs discussed in the main text, we report the energy-resolved hole–phonon and electron–phonon scattering rates in Fig. S5. Each point in the plot corresponds to scattering rate for an individual electron or hole state, with results given within 0.3 eV of the respective band edges. In addition to the total scattering rate, we show separately the contributions from LO and acoustic phonons. We find that the scattering rates are generally smaller in GaAs, consistent with the slower cooling dynamics in GaAs compared to GaN discussed in main text. In particular, the electron–acoustic-phonon scattering rates fall below the plotting range, with typical values of $\sim 10^{-4}$ fs^{-1}.

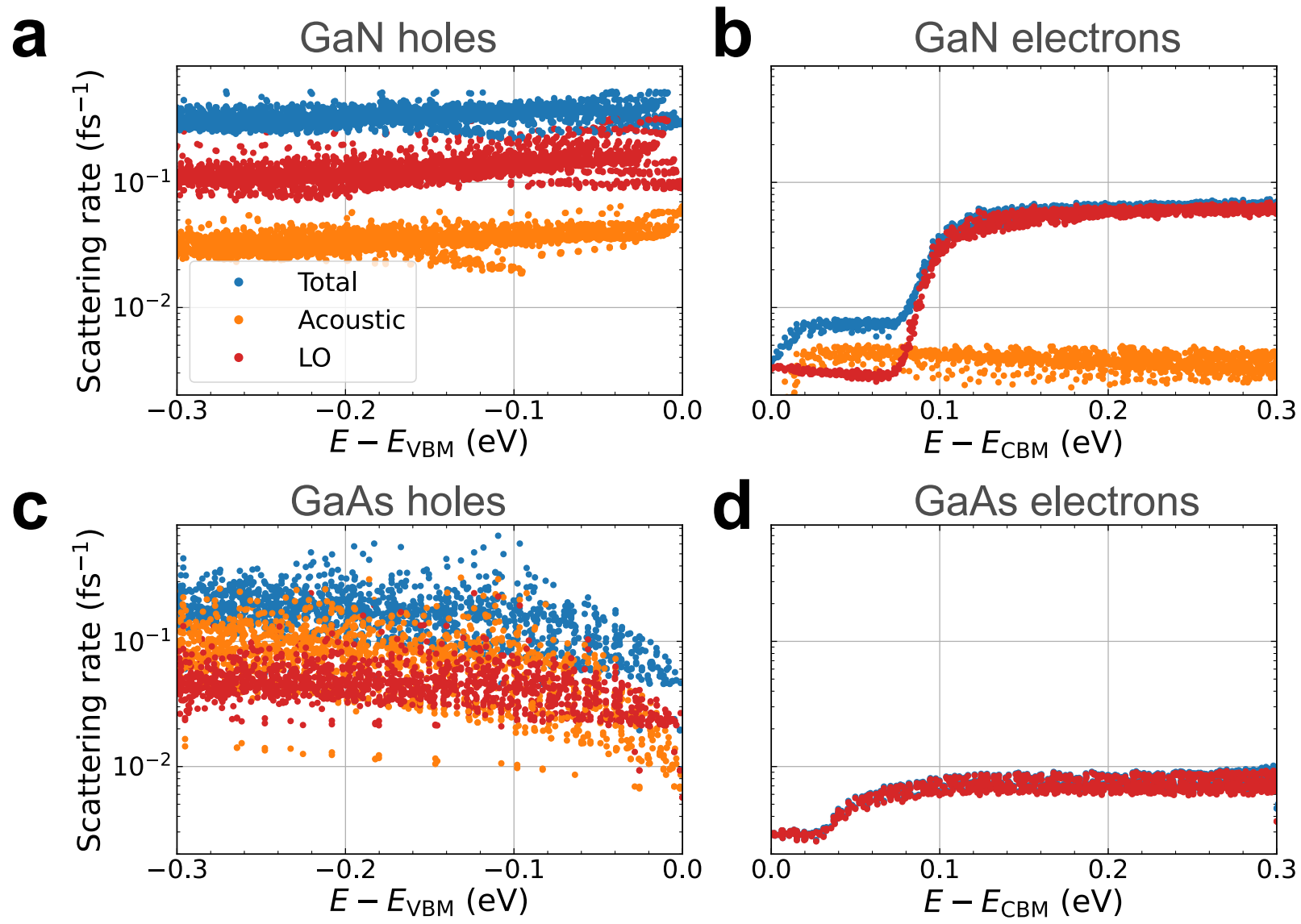

Fig. S5. **Scattering rates for holes and electrons in GaN and GaAs. a, b** Hole (**a**) and electron (**b**) scattering rates in GaN as a function of carrier excess energy relative to the band edge. **c, d** Corresponding hole (**c**) and electron (**d**) scattering rates in GaAs.
